# Outcomes of standalone ab interno trabeculotomy in the treatment of open-angle glaucoma in eyes with high myopia

**DOI:** 10.1186/s12886-023-03000-5

**Published:** 2023-06-12

**Authors:** Takeshi Yoshida, Takuhei Nomura, Sota Yoshimoto, Motohisa Ohno, Taiju Ito, Shintaro Horie, Kyoko Ohno-Matsui

**Affiliations:** 1 Department of Ophthalmology and Visual Science, Tokyo Medical and Dental University, Tokyo, Japan; 2 Department of Advanced Ophthalmic Imaging, Tokyo Medical and Dental University, 1-5-45 Yushima Bunkyo-Ku, Tokyo, 1138519 Japan

**Keywords:** High myopia, Pathological myopia, Trabeculotomy, Intraocular pressure, Open-angle glaucoma

## Abstract

**Background:**

We retrospectively evaluate the long-term efficacy and safety of trabeculotomy glaucoma surgery in treating open-angle glaucoma (OAG) in eyes with high myopia (HM).

**Methods:**

This study included 20 eyes with HM (axial length ≥ 26.5 mm) and OAG; age, preoperative IOP (intraocular pressure), and sex-matched 20 non-HM eyes (axial length < 26.5 mm) served as controls. Each eye underwent standalone ab interno trabeculotomy using a Kahook dual blade. A follow-up examination was performed 36 months after surgery. The main outcome measure was the operative success rate (i.e., a ≥ 20% pre- to post-operative reduction in IOP with or without IOP-lowering medication). Kaplan–Meier analysis was employed as a measure of surgical success. The secondary outcome measures were postoperative IOP, the number of glaucoma medications, and postoperative complications.

**Results:**

IOP and the number of glaucoma medications were statistically significantly reduced at all postoperative follow-up examinations. The Kaplan–Meier analysis demonstrated that the probability of postoperative success at 36 months was 45% and 65% for HM and non-HM eyes, respectively. In the HM group, the presence of pathological myopia was statistically significant risk factor for surgical failure. No critical postoperative complications were detected.

**Conclusions:**

In our study, the long-term efficacy of ab interno trabeculotomy in HM eyes with OAG was inferior to that in non-HM eyes with OAG. Our findings suggest that surgical indications for trabeculotomy in HM should be determined based on the presence of pathological myopia.

## Background

In high myopia (HM), the eyeball elongates excessively and the posterior tissue of the eyeball, including the retina, choroid, and sclera membranes, becomes extremely thin. Among HM, pathological myopia (PM) has attracted attention in terms of various ocular complications, such as retinal detachment, retinoschisis, macular holes, posterior staphyloma, myopic choroidal neovascularization, and open-angle glaucoma (OAG) [1–4]. Among the complications, OAG is becoming a serious concern because of severe visual function loss. However, the correlation between HM including PM and OAG has not been fully clarified to date. And the efficacy of IOP lowering surgery of glaucoma in HM including PM has not been fully understood, neither.

Trabeculotomy has proven to be a safe and effective procedure for lowering intraocular pressure (IOP) in patients with open-angle glaucoma (OAG) by incision of TM [5]. Recently, minimally invasive glaucoma surgery (MIGS) has been developed as a safer and less traumatic surgical intervention for glaucoma patients [6]. According to the commonly accepted definition, trabeculotomy using MIGS is a surgical procedure with an ab interno approach that causes minimal trauma (with very little or no scleral dissection), requires minimal or no conjunctival manipulation, and presents with a good safety profile and rapid recovery prospects. Thus, there has been growing interest in MIGS procedures and an increasing induction of trabeculotomy in recent years. However, this increase in surgical options remains to be supported by clear evidence of the efficacy of these procedures, and the efficacy and safety of trabeculotomy using MIGS in PM patients has not been fully elucidated.

The PM leads to structural changes in the posterior pole fundus of the eye, which can lead to loss of visual acuity. Recently an international panel of researchers in myopia uniformed classification system for PM [2, 4]. In the system (the META-PM classification), myopic maculopathy lesions are categorized into five categories from no myopic retinal lesions (category 0), tessellated fundus only (category 1), diffuse chorioretinal atrophy (category 2), patchy chorioretinal atrophy (category 3), to macular atrophy (category 4). Lacquer cracks, myopic neovascularization, and Fuchs spot were categorized as “plus signs”. Based on the classification, PM is defined as equal to or greater than the category 2, or presence of plus lesion, or the presence of a posterior staphyloma. In this manner, it is quite conceivable that the classification of PM may affect the efficacy of IOP lowering surgeries in OAG, however; there has been not investigated.

The most important known risk factor for OAG is elevated IOP, which occurs due to increased resistance to aqueous humor outflow within the conventional outflow pathway; the principal sites of outflow resistance in this pathway are the trabecular meshwork (TM) and Schlemm’s canal [7, 8]. Abnormalities in these sites among OAG patients have been demonstrated in many previous in vivo studies [9–11]. Moreover, a previous study showed that the morphology of the TM is likely to change in HM subjects. Chen et al. demonstrated that eyes with HM have a larger Schlemm's canal diameter and area, as well as a decreased trabecular meshwork thickness [12]. Such morphological abnormalities in the principal outflow site of the aqueous humor are considered to affect the surgical outcome of trabeculotomy in patients with HM, which might result in lower success rate of trabeculotomy in patients with HM compared to patients without HM. However, no studies have investigated the efficacy and safety of trabeculotomy glaucoma surgery in HM patients with OAG.

Therefore, the purpose of the present study was to evaluate the efficacy and safety of trabeculotomy surgery for the treatment of OAG in eyes with HM, as compared to those without HM. Moreover, we analyzed the characteristics of patients with HM who experienced either operative success or failure. We also analyzed the implication of the presence of PM with operative success in HM patients. This article presents a retrospective analysis of postoperative outcome measures, including changes in IOP, reductions in medication dependence, and postoperative complications.

## Methods

### Study design and population

We conducted a retrospective, non-randomized, interventional case series. This study was approved by the Ethics Committee of the Tokyo Medical and Dental University and conformed to the tenets of the Declaration of Helsinki and its later amendments. Due to the retrospective nature of this study, the requirement for informed consent was waived by the ethics review board. Data were collected from patient medical records of eyes with OAG and HM (axial length ≥ 26.5 mm) and age, sex and preoperative IOP matched non-HM controls (axial length < 26.5 mm) that underwent standalone ab interno trabeculotomy using a Kahook dual blade (KDB, New World Medical Inc, Rancho Cucamonga, CA, USA) at our medical center between April 2018 and December 2019. The surgeries were performed by two experienced surgeons (TY and TN). The indication for surgery was failure to control IOP despite administering maximally tolerated medical therapy. Only one eye was included for each patient. If two eyes had undergone surgery, the first eye that underwent surgery was chosen for evaluation in order to take advantage of the longer associated medical history. We obtained information on medical history, Goldmann applanation IOP (mmHg), axial length (mm), central corneal thickness (CCT; µm), number of glaucoma medications, and postoperative events and complications from patient medical records. PM discrimination was performed using the PM classification system, as described previously [4]. Inclusion criteria were as follows: mild to moderate OAG and age > 18 years. Exclusion criteria were as follows: patients with corneal opacities not permitting adequate ocular examinations, history of intraocular surgery without cataract surgery, and clinically significant peripheral synechiae/closed-angle glaucoma. KDB ab interno trabeculotomy was performed following a detailed discussion with the patient about the specific surgical indication and risks, and informed consent was obtained in all cases.

### KDB ab interno trabeculotomy

All surgeries were performed under topical anesthesia by two attending surgeons (TY and TN) specializing experienced in ab interno trabeculotomy. A KDB was used to incise the TM of the nasal angle in all surgeries. A Swan Jacob gonioprism lens (Ocular Instruments, Bellevue, WA, USA) was used to observe the angle opposite the corneal port, and a KDB was inserted into the anterior chamber through the corneal port. The KDB tip was then inserted into Schlemm's canal and moved circumferentially in order to incise the inner wall of the canal and the TM by approximately 120 degrees [13, 14].

### Follow-up

Postoperative follow-up visits were performed in our glaucoma clinic on Day 1, Week 1, Week 2, Month 1, Month 3, following which follow-up visits were performed every three months. Topical anti-glaucoma medication was prescribed if the patient’s IOP was high at a scheduled visit. Additional surgery was performed if the IOP was uncontrollable with maximally tolerated antiglaucoma medication. All follow-up visits included an assessment of the number and type of glaucoma medications and IOP measurements. In the present study, we collected data at Month 1, Month 3, Month 6, Month 12, Month 18, Month 24, and Month 36 following the surgery. These data were used for the statistical analysis described below.

### Outcome measures

The primary outcome measure in the present study was the operative success rate of the evaluated procedure. Success was defined as an IOP reduction of ≥ 20% with or without IOP-lowering topical medication that was maintained from Month 3 of follow-up onwards without the need for further glaucoma surgery. The secondary outcomes evaluated herein were pre- and postoperative IOP, number of glaucoma medications needed, CCT, and postoperative complications. Swept-source optical coherence tomography (SS-OCT) imaging (Triton, Topcon, Japan) and color fundus photographs were used for the diagnosis and classification of posterior staphyloma. Safety was assessed by calculating the proportion of patients with postoperative complications, including transient hypotony, hyphema, IOP spikes (IOP ≥ 26 mmHg), shallow or flat anterior chamber, infection, and expulsive haemorrhage. Hyphema, defined as presence of blood in the anterior chamber, was reported as complication only when requiring surgical washout, either because the hyphema did not resolve within the first two weeks of surgery or there was a complete hyphema. IOP spikes were only considered a complication when these spikes presented during two consecutive visits and lasted for at least two weeks. Transient hypotony was defined as an IOP of < 6 mmHg on two consecutive visits occurring more than two weeks apart or when low IOP was accompanied by maculopathy, a shallow or flat anterior chamber, or choroidal effusions/hemorrhage.

### Statistical analysis

All statistical analyses were performed using SPSS for Windows (IBM Corp., Armonk, NY, USA). Comparisons of clinical characteristics between the HM and non-HM groups were performed using the Mann–Whitney U test for continuous variables and Fisher’s exact test for categorical variables. A Kaplan–Meier survival curve analysis was used to evaluate the cumulative probability of success, and the log-rank test was used to compare outcomes between the HM and non-HM groups. The following factors were tested for associations with surgical failure: sex, age, preoperative IOP, CCT, the presence of pathological myopia, and axial length. A *P*-value of < 0.05 was considered the threshold for statistical significance. All statistical values are presented as means ± standard deviations (SD).

## Results

### Patients’ data

Patients’ clinical and demographic characteristics are provided in Table 1. Ab interno trabeculotomy using KDB was performed in 20 HM patients (20 eyes) with OAG and in 20 non-HM patients (20 eyes) with OAG. The male-to-female ratio was 7:13 in the HM group and 10:10 in the non-HM group (*P* = 0.220). The mean age of the HM group was 58.4 ± 19.4 years (range, 35 to 81 years), and the mean age of the non-HM group was 65.6 ± 17.1 years (range, 40 to 85 years) (*P* = 0.201). The mean axial lengths of the HM and non-HM groups were 30.1 ± 2.6 mm (range, 26.5 to 38.3 mm) and 24.8 ± 1.1 mm (range, 24.8 to 26.1 mm), respectively (*P* < 0.01), the mean CCT values in the HM and non-HM groups were 525.0 ± 37.2 µm (range, 477 to 583 µm) and 518.2 ± 47.6 µm (range, 445 to 607 µm), respectively (*P* = 0.763), and the respective mean IOP values were 24.2 ± 6.4 mmHg (range, 15 to 34 mmHg) and 22.6 ± 8.1 mmHg (range, 15 to 34 mmHg), respectively (*P* = 0.903). The mean numbers of glaucoma medications in the HM and non-HM groups were 4.5 ± 0.6 (range, 3 to 5) and 4.4 ± 1.1 (range, 1 to 5), respectively (*P* = 0.934). PM was present in 11 of 20 eyes in the HM group and in 0 of 20 eyes in the non-HM group (*P* < 0.01). Patients’ recorded history of previous ocular surgery included cataract surgery in eight patients in the HM group and in five patients in the non-HM group. All eyes in the present study underwent standalone trabeculectomy using KDB.

**Table 1:** Patient characteristics

Preoperative data	HM (*n* = 20)	non-HM (*n* = 20)	*P*-value
Sex (Male/Female)	7/13	10/10	0.220*
Age (years)	58.4 ± 19.4	65.6 ± 17.1	0.201**
Axial length (mm)	30.1 ± 2.6	24.8 ± 1.1	< 0.01**
CCT (µm)	525.0 ± 37.2	518.2 ± 47.6	0.763**
PM Grade 0	0	20	< 0.01*
Grade 1	9		
Grade 2	5		
Grade 3	4		
Grade 4	2		
IOP (mmHg)	23.9 ± 6.6	23.7 ± 6.5	0.903**
Medication number	4.5 ± 0.6	4.4 ± 1.1	0.934**

*HM* High myopia, *CCT* Central corneal thickness, *IOP* Intraocular pressure, PM Pathological myopia, Mean ± SD, *P*-value: * Fisher's exact test, **Mann–Whitney’s U test

### Postoperative data

Postoperative IOP changes are shown in Table 2. At 1, 3, 6, 12, 18, 24 and 36 months following trabeculotomy, average IOP values in the HM and non-HM groups were as follows: 16.6 ± 3.2 vs. 15.0 ± 3.5 mmHg, 15.7 ± 4.4 vs. 15.2 ± 3.3 mmHg, 15.9 ± 4.8 vs.14.5 ± 3.1 mmHg, 14.3 ± 2.8 vs. 15.3 ± 3.1 mmHg, 14.5 ± 2.4 vs. 15.2 ± 2.5 mmHg, 14.9 ± 2.8 vs. 15.1 ± 2.9 mmHg, and 14.5 ± 3.7 vs. 15.2 ± 2.7 mmHg, respectively. IOP was statistically significantly decreased at each postoperative follow-up examination as compared with baseline in the HM and non-HM groups (*P* < 0.001). And there were no statistically significant differences between the two groups at each postoperative follow-up examination.

**Table 2:** The change of IOP after trabeculotomy

	Baseline	1 M	3 M	6 M	12 M	18 M	24 M	36 M
HM	24.2 ± 6.4	16.6 ± 3.2*	15.7 ± 4.4*	15.9 ± 4.8*	14.3 ± 2.8*	14.5 ± 2.4*	14.9 ± 2.8*	14.5 ± 3.7*
non-HM	22.6 ± 8.2	15.0 ± 3.5*	15.2 ± 3.3*	14.5 ± 3.1*	15.5 ± 3.1*	15.2 ± 2.5*	15.1 ± 2.9*	15.2 ± 2.7*

^*^ Paired T test *P* < 0.05

Postoperative glaucoma medication number changes are presented in Table 3. At 1, 3, 6, 12, 18, 24 and 36 months after undergoing trabeculotomy, the average number of glaucoma medications in the HM and non-HM groups were as follows: 1.1 ± 1.6 vs. 1.5 ± 1.7, 2.4 ± 2.1vs. 2.4 ± 1.6, 2.5 ± 2.0 vs. 3.1 ± 1.5, 2.2 ± 2.0 vs. 3.1 ± 1.5, 2.0 ± 2.2 vs. 3.1 ± 1.6, 1.7 ± 2.3 vs. 3.1 ± 1.6, and 2.3 ± 2.1 vs. 3.1 ± 1.7, respectively. The number of glaucoma medications decreased statistically significantly at each postoperative follow-up examination (as compared with baseline medication profiles) in the HM and non-HM groups (*P* < 0.01). And there were no statistically significant differences between the two groups at each postoperative follow-up examination.

**Table 3:** The change of glaucoma medication number after trabeculotomy

	Baseline	1 M	3 M	6 M	12 M	18 M	24 M	36 M
HM	4.5 ± 0.6	1.3 ± 1.6*	2.3 ± 2.2*	2.3 ± 2.1*	2.3 ± 2.1*	2.1 ± 2.0*	2.0 ± 2.2*	2.1 ± 2.0*
non-HM	4.4 ± 1.1	1.5 ± 1.7*	2.4 ± 1.6*	3.1 ± 1.5*	3.1 ± 1.5*	3.1 ± 1.6*	3.1 ± 1.6*	3.1 ± 1.7*

^*^ Paired T test *P* < 0.05

Postoperative complications are shown in Table 4. No incidents of postoperative transient hypotony, hyphema requiring surgical washout, shallow or flat anterior chamber, infection, or expulsive haemorrhage were observed during the follow-up period in either group. Transient IOP spikes (IOP ≥ 26 mmHg) were seen in 5 eyes (25%) in the HM group and in 7 eyes (35%) in the non-HM group. The incidence of postoperative complication has no significant difference between the two groups (*P* = 1.0). Finally, 7 eyes (35%) in the HM group and 4 eyes (20%) in the non-HM group underwent additional trabeculectomy glaucoma surgery because of uncontrolled IOP.

**Table 4:** Postoperative complications

Complications	HM (*n* = 20)	non-HM (*n* = 20)	*P* value
Hyphema	0	0	*P* = 1.0*
Hypotony	0	0	
Shallow or flat AC	0	0	
Hypertony	5	7	
Infection	0	0	
Expulsive hemorrhage	0	0	
Additional glaucoma surgery	7	4	

*HM *High myopia, *AC* Anterior chamber, *Mann–Whitney’s U test

**Fig. 1:**
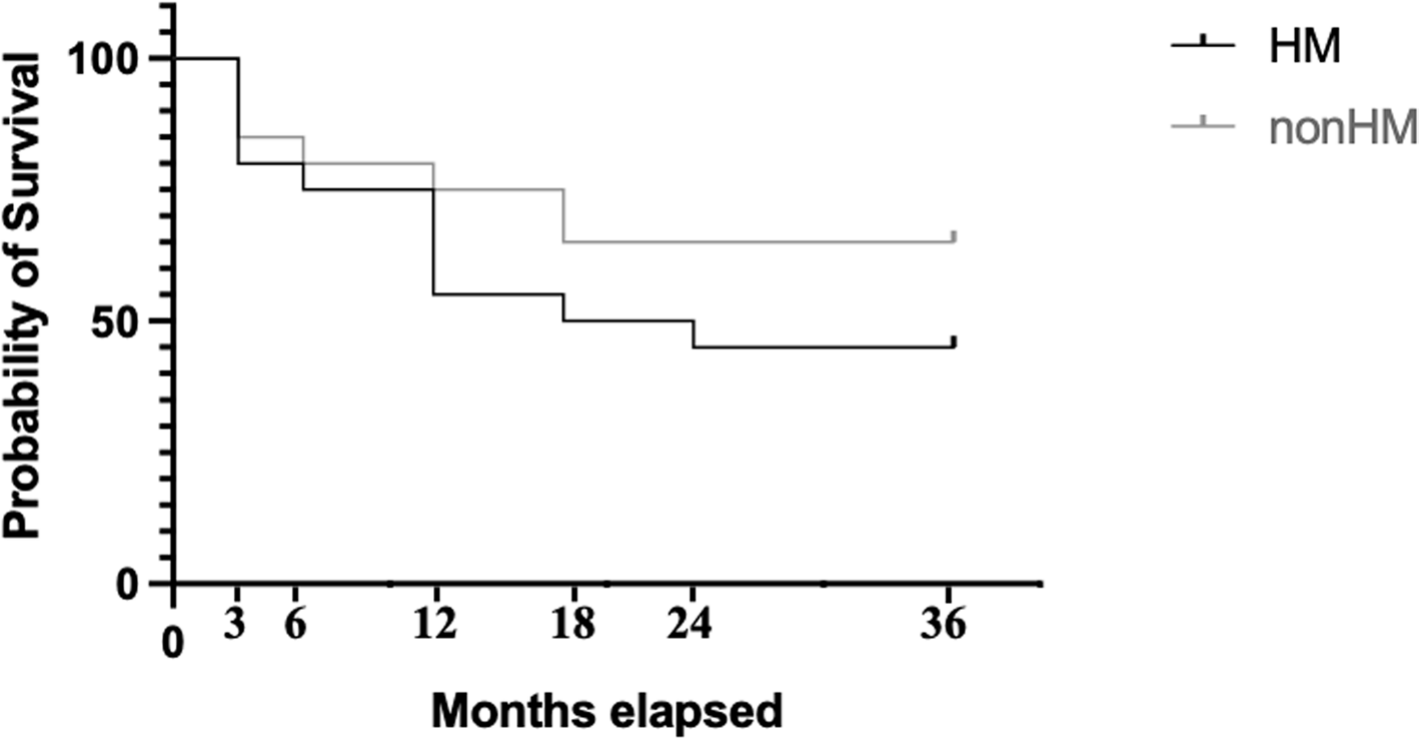
Survival curve. The probability of surgical success was defined as an IOP (intraocular pressure) reduction of ≥ 20% as compared with baseline, with or without the need for IOP lowering topical medication. This probability was calculated using a Kaplan–Meier life-table analysis. The black line represents HM (high myopia) patients, and the gray line represents non-HM patients. Statistically significant difference was found between the two groups (*P* = 0.038; log-rank test)

Figure 1 shows the Kaplan–Meier survival curve analysis for the HM and non-HM groups. At 3, 6, 12, 18, 24, and 36 months after undergoing trabeculotomy, the probabilities of surgical success in the HM and non-HM groups were as follows: 85 vs. 70%, 80 vs. 75%, 75 vs. 45%, 65 vs. 35%, 65 vs. 35%, and 65 vs. 30%, respectively. A significant (*p* = 0.038) difference was seen in the survival probabilities between eyes with HM and those without HM over the course of follow-up.

In Table 5, we present characteristics at baseline with respect to success and failure in the HM group at 36 months postoperatively. The male-to-female ratio was 5:4 in the success group and 2:9 in the failure group in HM patients (*P* = 0.160). The respective average age in the success and the failure groups were 54.2 ± 23.7 and 61.8 ± 15.3 years (*P* = 0.564), the respective average axial lengths in the success and the failure groups were 29.2 ± 1.8 and 30.8 ± 3.0 mm (*P* = 0.157), the respective average IOP values were 23.9 ± 8.2 and 23.7 ± 6.0 mmHg (*P* = 0.779), the respective average CCT in the success and the failure groups were 524.6 ± 36.1 and 527.6 ± 40.6 µm (*P* = 0.808),and the respective average numbers of glaucoma medications were 4.7 ± 0.5 and 4.3 ± 0.7 (*P* = 0.265). PM was present in 2 eyes of 9 eyes in the success group and in 9 eyes of 11 eyes in the failure group (*P* = 0.022). We found that only the presence of PM at baseline affected surgical outcomes in HM patients with OAG.

**Table 5:** Patient characteristics of success and fails in high myopia at 36 months

Preoperative data	Success (*n* = 9)	Failure (*n* = 11)	*P*-value
Sex (Male/Female)	5/4	2/9	0.160*
Age (years)	54.2 ± 23.7	61.8 ± 15.3	0.564**
Axial length (mm)	29.2 ± 1.8	30.8 ± 3.0	0.157**
CCT (µm)	524.6 ± 36.1	527.6 ± 40.6	0.808**
PM (yes/no)	2/7	9/2	0.022*
Medication number	4.7 ± 0.5	4.3 ± 0.7	0.265**
IOP (mmHg)	25.7 ± 7.7	23.6 ± 5.2	0.779**

*CCT* Central corneal thickness, *PM* Pathological myopia

*P*-value::*Fisher exact test **Mann–Whitney’s U test

## Discussion

To the best of our knowledge, this is the first study evaluating surgical outcomes in trabeculotomy in patients with HM and OAG. We evaluated the postoperative success rate and outcomes in eyes with HM that were subjected to ab interno trabeculotomy. We detected a statistically significant cumulative probability of failure following trabeculotomy in HM eyes as compared to those in non-HM eyes, the success rate was 65% in the non-HM group at 36 months after surgery, whereas the success rate in the HM group was only 45%. And we identified that the presence of PM at baseline were important risk factors for surgical failure in HM patients with OAG. As MIGS has gained increasing attention among glaucoma specialists due to its safety profile and the ease of the technique, the present study provides important insights suggesting that trabeculotomy (and possibly outflow channel surgeries in general) have intrinsic limitations that should be considered in the surgical management of glaucoma in patients with HM.

Many population-based studies have shown that the prevalence of OAG increases with increasing myopia, and that the association with glaucomatous optic neuropathy is more pronounced in patients with moderate-to-high myopia [15, 16]. Numerous studies have likewise shown that refractive errors, especially medium-to-high myopia, are independent risk factors for OAG [15, 17, 18]. The reason myopic eyes (especially HM eyes) appear to be more susceptible to glaucomatous damage when compared with non-myopic eyes is unclear. However, we note that high myopia is an extreme form of myopia that causes excessive eyeball elongation. In turn, excessive elongation of the eyeball induces strong mechanical stress on all eye tissues, and this stress is associated with many severe comorbidities (such as retinal detachment, subretinal neovascularization, macular degeneration, and glaucoma) [19]. Recently, we showed that the increased fragility of the lamina cribrosa due to mechanical stress results in the development of glaucomatous visual field defects in patients with HM [20], which also indicates that excessive eyeball elongation may be a crucial risk factor in the development of glaucoma in HM patients. Chen et al. previously reported that HM patients have a larger Schlemm’s canal diameter and area as well as a decreased trabecular meshwork thickness [12] resulting from eyeball elongation. Schlemm's canal is a ring structure responsible for maintaining fluid homeostasis in the anterior chamber of the eye by draining the aqueous humor from the TM into the collecting channel. Obstruction of the aqueous humor flow in this conventional pathway leads to IOP elevation in glaucoma, which may in turn lead to poor outcomes in trabeculotomy. In addition, Fea et al. recently reported patients with HM were at higher risk of hypotony-related complications after trabeculectomy because of thinner sclera [21].Thus, excessive eyeball elongation induces many morphological changes in HM eyes; these changes are responsible for the development of ocular disease and may affect treatment results.

According to the findings of previous studies, the level of surgical success for this procedure (typically defined as an IOP of ≤ 21 with a 20% decrease in IOP following standalone trabeculotomy surgery using MIGS) ranges from 45 to 60% [22–26]. Kaplowitz et al. alternatively defined surgical success as an IOP of ≤ 21 with a 20% postoperative decrease in IOP while avoiding reoperation; using this definition, the success rate for trabeculotomy using the Trabectome procedure was 46 ± 3.4% two years postoperatively [22]. In addition, Gosling et al. defined surgical success as an IOP reduction of > 20% (IOP < 21 mm); these researchers showed that, given this definition, the success rate for trabeculotomy using TrabEX® was 54% at 25 months postoperatively [23]. The success rate of non-HM with OAG in the present study has good agreement with these previous studies. And we demonstrated that subjects with HM had significantly poor trabeculotomy surgery outcomes (45%) as compared with non-HM subjects (65%). To our knowledge, no previous studies have investigated long-term outcomes of trabeculotomy in HM patients. In the present study, our result become important evidence in a decision of applying trabeculotomy in HM patients. We speculate that poor surgical outcomes in HM patients may have several causes. First, HM is characterized by an above-normal axial length [19]; this elongation may be accompanied by an enlarged Schlemm’s canal diameter, as described previously [12]. Moreover, we note that intrascleral collector channels and the deep scleral plexus course through the sclera to the episcleral veins [27]; deformed and irregular eye shapes in HM may cause lesions and obstruction in intrascleral collector channels, leading to increased resistance distal to Schlemm’s canal and finally resulting in the expansion of Schlemm’s canal. Especially in HM eyes with PM, the deformation of eyeball is considered severe, and their sclera may be structural altered. Previous investigations have demonstrated marked thinning of the sclera, choroid, and retina in HM with stretching of the sclera, choroid, and retina caused by axial length elongation [28–30]; this may result in a disordered presentation of the intra-scleral collector channels, deep scleral plexus, and epi-scleral veins. Second, HM patients experience a series of collagen fiber changes [31] including a predominantly laminar collagen fiber bundle arrangement, a loss of fiber cross-links, and a reduction in collagen and glycosaminoglycan synthesis. Presumably, these changes increase the plasticity of collagen fibers and reduce their cross-linking stability. Changes in collagen fibers in HM (i.e., with regard to structure, biochemistry, and biomechanical properties around Schlemm’s canal) may lead to the obstruction of aqueous humor outflow into intra-scleral collector channels; these changes might also induce a functional failure in intra-scleral collector channels, the deep scleral plexus, and the epi-scleral veins. Third, recent studies have indicated that many metabolites and cytokines, such as interleukin-6 and matrix metalloproteinase-2 (known inflammatory factors in the aqueous humor), are statistically significantly altered in patients with HM [32]. This type of dysfunction in intraocular fluid could contribute to the formation of scars around the surgical area of the TM, which may inhibit aqueous humor outflow into collector channels and may contribute to the lower surgical success rate seen in HM. These findings require additional investigation. Relevant histological studies would help answer these questions more comprehensively.

In the present study, we also demonstrated PM to be a risk factor for failure of trabeculotomy in HM patients. PM differs distinctly from HM with regard to a range of parameters. For example, high myopia is characterized by a high degree of myopic refractive error, whereas PM is defined by the presence of typical complications in the posterior fundus. Refractive error or axial length alone often does not adequately reflect PM [33]. Indeed, the AXL was not statistical risk factor for trabeculotomy in HM patients in the present study. In the META-PM classification, PM is defined according to a categorization that is equal to or greater than myopic maculopathy (Category 2) [2]. In the current study, we demonstrated 11 eyes showed PM among the 20 eyes, and nine in the 11 eyes exhibiting surgical failure in HM patients with OAG. This high ratio of PM in the surgical failure group is remarkable. In higher grade of PM, the structure of retina is dramatically altered, and it is possible that the ocular tissues abnormality extends to the sclera. In HM eyes, several scleral abnormalities such as thinning of sclera, posterior staphyloma and myopic scleral pit, have been described [34, 35], and may influence aqueous humor outflow, especially in the PM eyes. Taken together, it is reasonable our results that the outcome of trabeculotomy in PM patients was not favorable. We should pay attention the presence of PM before trabeculotomy surgery in HM patients. The presence of PM may be the most important factors predicting trabeculotomy outcomes in HM patients.

A limitation of the present study is that the included data were obtained from a relatively small number of eyes. However, this is the first to identified 36 months standalone ab interno trabeculotomy outcome in patients with HM, and relatively few OAG patients with extremely long axial length were included in our study (mean axial length, 30.1 mm), which may support future study. We also note that a longer follow-up study is needed for the long-term evaluation of glaucoma surgery outcomes and that, in general, large-scale and long-term studies are needed to confirm our findings more definitively. However, we preliminarily conclude that the long-term efficacy and safety of ab interno trabeculotomy in HM eyes with OAG may be inferior to that in non-HM eyes with OAG.

## Conclusions

Our findings suggest that surgical indications for trabeculotomy should be determined based on the presence of PM in addition to HM, which may be important to avoid unnecessary or low effective surgery. Our findings could indicate future research directions and improve the indications criteria for surgical treatment of glaucoma.

## Data Availability

The datasets used and/or analyzed during the current study are available from the corresponding author on reasonable request.

## Abbreviations

OAG: Open-angle glaucoma
IOP: Intraocular pressure
HM: High myopia
KDB: Kahook dual blade
PM: Pathological myopia
TM: Trabecular meshwork
MIGS: Minimally invasive glaucoma surgery
CCT: Central corneal thickness
